# Genome-wide diversity, population structure and signatures of inbreeding in the African buffalo in Mozambique

**DOI:** 10.1186/s12862-024-02209-2

**Published:** 2024-03-04

**Authors:** Paolo Colangelo, Marika Di Civita, Carlos M. Bento, Paolo Franchini, Axel Meyer, Nadiya Orel, Luis C. B. G. das Neves, Fernando C. Mulandane, Joao S. Almeida, Gabriele Senczuk, Fabio Pilla, Simone Sabatelli

**Affiliations:** 1 National Research Council, Research Institute on Terrestrial Ecosystems, Via Salaria km 29.300, 00015 Montelibretti (Roma), Italy; 2 Department of Agricultural, Environmental and Food Sciences, University of Molise, 86100 Campobasso, Italy; 3 Department of Biology and Biotechnologies “Charles Darwin”, Sapienza University, Viale dell’Università 32, 00185 Roma, Italy; 4 Natural History Museum, Eduardo Mondlane University, Travessia do Zambeze 104, 1100 Maputo, Mozambique; 5 Department of Biology, University of Konstanz, Konstanz, Germany; 6 Department of Ecological and Biological Sciences, University of Tuscia, Viale dell’Università s.n.c, 01100 Viterbo, Italy; 7 Biotechnology Centre of Eduardo Mondlane University, Maputo, Mozambique; 8 Department of Veterinary Tropical Diseases, Faculty of Veterinary Sciences, University of Pretoria, Pretoria, South Africa; 9 Mozambique wildlife alliance, Maputo, Mozambique

**Keywords:** RAD-seq, Population genomics, Homozygosity, Admixture, Gene flow, *Syncerus caffer caffer*

## Abstract

The African buffalo, *Syncerus caffer*, is a key species in African ecosystems. Like other large herbivores, it plays a fundamental role in its habitat acting as an ecosystem engineer. Over the last few centuries, African buffalo populations have declined because of range contraction and demographic decline caused by direct or indirect human activities. In Mozambique, historically home to large buffalo herds, the combined effect of colonialism and subsequent civil wars has created a critical situation that urgently needs to be addressed. In this study, we focused on the analysis of genetic diversity of *Syncerus caffer caffer* populations from six areas of Mozambique. Using genome-wide SNPs obtained from ddRAD sequencing, we examined the population structure across the country, estimated gene flow between areas under conservation management, including national reserves, and assessed the inbreeding coefficients. Our results indicate that all studied populations of *Syncerus caffer caffer* are genetically depauperate, with a high level of inbreeding. Moreover, buffaloes in Mozambique present a significant population differentiation between southern and central areas. We found an unexpected genotype in the Gorongosa National Park, where buffaloes experienced a dramatic population size reduction, that shares a common ancestry with southern populations of Catuane and Namaacha. This could suggest the past occurrence of a connection between southern and central Mozambique and that the observed population structuring could reflect recent events of anthropogenic origin. All the populations analysed showed high levels of homozygosity, likely due to extensive inbreeding over the last few decades, which could have increased the frequency of recessive deleterious alleles. Improving the resilience of *Syncerus caffer caffer* in Mozambique is essential for preserving the ecosystem integrity. The most viable approach appears to be facilitating translocations and re-establishing connectivity between isolated herds. However, our results also highlight the importance of assessing intraspecific genetic diversity when considering interventions aimed at enhancing population viability such as selecting suitable source populations.

**Supplementary Information:**

The online version contains supplementary material available at 10.1186/s12862-024-02209-2.

## Introduction

Since the 19th century, many African mammal species have experienced a decline due to human population expansion, overexploitation, habitat loss and degradation, wars, and the spread of livestock-borne diseases [1–2]. Currently, several populations of endangered species survive in protected areas that are often poorly connected, not ensuring a sufficient gene flow among isolated populations [3].

Like other mammal species, the African buffalo (*Syncerus caffer*) has experienced shifts in its distribution range since the last phases of the Pleistocene, resulting in the expansion and contraction of suitable habitat [4–5]. These changes have left a distinctive signature in its genome. However, over the past 10,000 years, there has been evidence of a drop in the effective population size (N_e_) of *S. c. caffer* populations, coinciding with the increase in human populations [6]. Moreover, during the last two centuries, *S. caffer* has undergone a significant numerical reduction and shrinking of its distribution range due to war, disease, poaching [7], and expansion of livestock activity [8]. This species is now listed as “Near Threatened” due to population decline over extensive areas [9] (IUCN 2019). In Mozambique, where the nominal subspecies *S. c. caffer* is present, after the outbreak of civil war (1976–1992), the African buffalo populations experienced a significant decline both within and outside protected areas [10]. For instance, in the emblematic Gorongosa National Park, no buffalo were sighted during aerial counts in 2000, with the surviving population estimated to be less than 100 individuals at that time. This period also witnessed a decline of over 90% in the populations of large mammals [10–11]. Although the biomass of large mammalian herbivores in the country has substantially recovered since 1994, the species composition has shifted dramatically, with formerly dominant large herbivores, including elephant (*Loxodonta africana*), hippo (*Hippopotamus amphibius*), buffalo (*Syncerus caffer*), zebra (*Equus quagga*), and wildebeest (*Connochaetes taurinus*), now outnumbered by waterbuck (*Kobus ellipsiprymnus*) and other small to mid-sized antelopes [11]. However, large and megaherbivores play a crucial functional role in terrestrial ecosystems by acting as ecosystem engineers [12–13]. They prepare the habitat for small and mid-sized herbivores [14–16], as well as mitigate the impact of large uncontrolled fires [17]. Thus, a reduction in the number of large herbivores could have an enormous ecological cost [12]. Therefore, the decline of *S. caffer* populations in many areas of Africa is particularly concerning as it has negative implications for the whole savanna ecosystem.

Between 2006 and 2011 the original buffalo population of Gorongosa was reinforced with 186 individuals translocated from the Kruger National Park in South Africa and the adjacent Limpopo National Park in Mozambique (Lopes Pereira, pers. Com.) in an attempt to increase the genetic diversity of *S. c. caffer* in the iconic Gorongosa National Park. Translocations are rarely followed up by monitoring activities, which means that the outcomes of these efforts are often unknown, and the eventual causes of failures are rarely understood [18]. While measuring an increase in population growth and size is often used to judge the success of reintroduction programs [19], it is important to note that the ability of a population to persist in the long term is also strongly influenced by the levels of genetic diversity. Therefore, simply achieving a demographic increase does not necessarily mean that genetic diversity has been restored, which is indispensable for the long-term persistence of the target populations [20].

Despite the clear and urgent necessity of planning actions to restore the buffalo population in Mozambique, including translocations, there is a lack of information regarding the current status of relict herds, particularly in terms of connectivity and genetic diversity. Furthermore, it is important to avoid genetic dilution during the process of translocation, as this carries the risk of losing the best characteristics of both the original and receiving populations. Therefore, the biogeographical aspects of populations must be considered before initiating the translocation process.

In the last decades, genetic assessments and monitoring are increasingly used for wildlife conservation and management [21–23]. Particularly, population genomics has provided key insights into ecological and evolutionary processes in natural and managed populations [24]. Population genomic data provide new opportunities for detecting natural selection and quantifying adaptive genetic variation in natural populations [25], while simultaneously increasing the accuracy and precision of estimates of genome-wide diversity [26], population structure, and demographic parameters [27].

The overarching aim of this research is to estimate the genetic diversity of African buffaloes in Mozambique, and to evaluate the population structure and connectivity among different areas using restriction-site-associated DNA (RAD) sequencing. Among the molecular methods aimed at genotyping a dense set of markers in a large set of individuals, RAD sequencing has become the preferred method of choice in molecular ecology and conservation studies [28, 29]. The RAD-based approaches are particularly valuable and widely used when applied to non-model organisms, where screening a large number of SNPs (Single Nucleotide Polymorphisms) might be cost- and time-prohibitive.

Despite the critical role of African buffaloes in Mozambique, there has been limited research that included individuals from this area [7, 30]. More recent studies that employ new genomic tools also did not include individuals from Mozambique [4, 5]. Therefore, in this study, we used genome-wide data of *S. c. caffer* to gain a deeper understanding of the population structure and connectivity between protected areas in Mozambique and to have an insight on the level of inbreeding of the focal populations with special attention to Gorongosa National Park buffaloes. Our emphasis on Gorongosa NP is due to the dramatic bottleneck experienced by this protected area and the recent restocking program. To our knowledge, there has been no previous assessment of the pre-restocking genetic diversity of African buffaloes in this area. Comparing the genetic diversity and inbreeding coefficients of the original Gorongosa population to other areas of Mozambique can provide a baseline for evaluating the effectiveness of current conservation efforts and assisting national and local authorities in developing effective conservation policies in Mozambique. An excessive numerical reduction and the consequent inbreeding are expected to increase the occurrence of recessive alleles, potentially making buffalo populations more vulnerable to environmental stressors [31]. Therefore, effective management of buffalo populations is critical for the survival of the species in Mozambique, and genetic monitoring is widely recognized as an essential tool to inform intervention plans [22].

## Materials and methods

### Sample collection and ethics statement

The blood samples used in this study were collected during past veterinary operations within the Gorongosa National Park (research permit: PNG/DSCi/C156/2019) and from animals immobilized under the mandate granted by the National Administration of Conservation Areas (ANAC) to the vet team of the Mozambique Wildlife Alliance (MWA). Samples are currently preserved at the Natural History Museum of Maputo (NHM-UEM) and the Biotechnology Centre of the Eduardo Mondlane University (CB-UEM) BioBank.

**Fig. 1:**
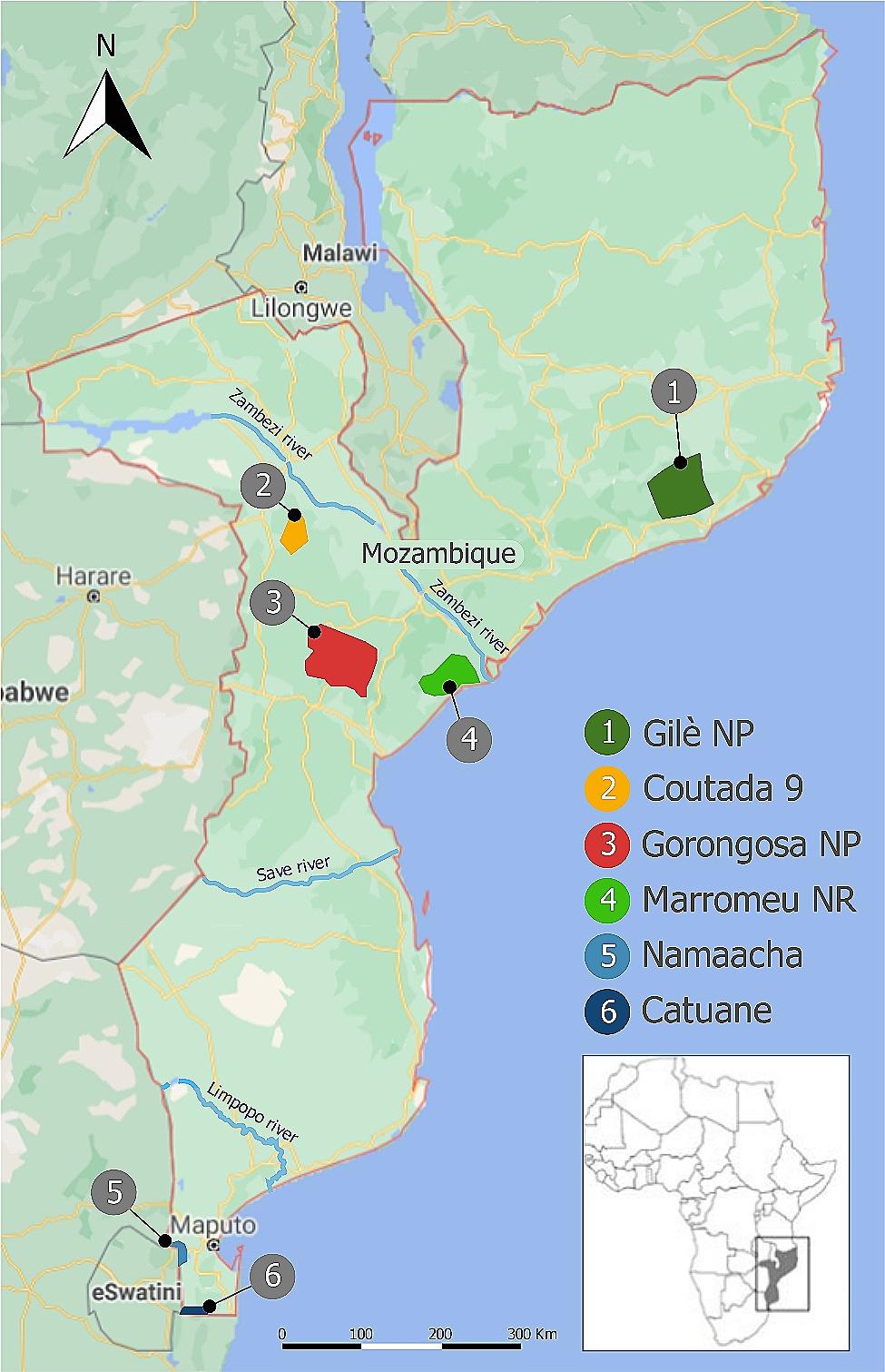
Map of Mozambique showing the six sampling localities of the African Buffalo (*Syncerus c. caffer*) analysed in this study. The map was created by utilizing a Google Maps image as a template (https://www.google.com/maps/place/Mozambico) and modified using Adobe Illustrator v2021 to highlight the sampling areas

A total of 70 *S. c. caffer* blood samples, stored in ethylenediaminetetraacetic acid (EDTA), were collected from six localities: four protected areas of Central Mozambique (Gorongosa National Park, Marromeu National Reserve, Gilè National Park, and Coutada 9) and two in free areas in the south of Mozambique (Namaacha and Catuane). Details of sampling localities are shown in Fig. 1 and in Supplementary Table S1. Genomic DNA extraction was performed in the Natural History Museum of Maputo (NHM-UEM) and the Biotechnology Centre (CB-UEM) laboratories. The DNeasy Blood & Tissue Kit (Qiagen, Valencia, USA) was used for DNA extraction following the manufacturer’sguidelines. The DNA extracts were stored at − 80 °C in the NHM-UEM and CB-UEM Biobank.

### Library preparation and sequencing

Sequencing libraries were constructed using the quaddRAD protocol developed by [32]. This protocol follows the general principles of the original double-digest RAD-Seq (ddRAD) approach developed by [33], with modifications that allow marking PCR duplicates, increasing multiplexing capacity, and minimise hands-on time. Genomic DNA concentration and quality were assessed using a Qubit v4.0 fluorometer (Life Technologies, Darmstadt, Germany) and a Bioanalyzer 2100 (Agilent Technologies, Palo Alto, USA), respectively. Approximately 200 ng of DNA per individual sample was digested using the restriction enzymes PstI (rare cutter) and MspI (frequent cutter). A single quaddRAD library including 70 barcoded individuals was size-selected from 450 to 550 bp in a Pippin Prep system (Sage Science, Beverly, USA) and paired-end sequenced (2 × 150 bp) in an Illumina HiSeq X-Ten platform at the Beijing Genomics Institute (BGI, Hong Kong).

### Sequence alignment and genotyping

PCR clone removal, quality filtering, and demultiplexing of raw sequence data were performed using the *clone_filter* and *process_radtags* modules implemented in the Stacks v2.59 package [34]. Clean sequences of each individual were independently aligned to the *S. caffer* reference genome (NCBI GenBank assembly accession: GCA_902825105.1) by using the Burrows-Wheeler Aligner bwa-mem v0.7.17 [35]. Variant calling was carried out using the Stacks’s *gstacks* program using default settings. Once the loci have been identified and genotyped, *gstacks* phases the SNPs at each locus for each individual into a series of haplotypes using a Bayesian algorithm [36, 37].

In a second step, the output assembled by *gstacks* was processed by the Stacks’s *Populations* program that processes the dataset based on various parameters. Specifically, rare variants, often resulting from PCR artifacts, were removed by setting a minor allele frequency (MAF) of 5% (--min-maf 0.05). The maximum observed heterozygosity required to process a nucleotide site was set to 70% (--max-obs-het 0.70) and all variants exceeding this heterozygosity threshold were eliminated; only the alleles present in at least 90% of the individuals of the total sample were retained (-R 0.9). The resulting dataset included 100,646 variants. To reduce the effect of non-independence of markers due to physical linkage and linkage disequilibrium (LD), and thus satisfying one of the main assumptions of model-based algorithms used in this study, a second dataset was produced by PLINK 1.9 to filter for LD the original dataset (parameters: --indep-pairwise 50 5 0.2), resulting in a new dataset including 53,056 variants.

### Population structure, admixture and gene flow

For each population, we used Stacks to estimate standard diversity indexes such as heterozygosity, homozygosity and nucleotide diversity (π), and to assess population differentiation measured with F_st_ and D_xy_. Population structure and admixture were estimated using the dataset pruned for LD. We computed a principal component analysis (PCA) using the R package SNPRelate v1.30.1 [38], whereas we estimated individual ancestry and admixture using the software Admixture v1.3.0 [39, 40]. Admixture uses a maximum likelihood estimation of individual ancestries from multilocus SNP genotype datasets. The best number of underlying populations was assessed by using the cross-validation procedure implemented in Admixture. A good value of K, i.e. the number of predefined genetic clusters or ancestral populations, is expected to exhibit a low cross-validation error compared to other K values. We then tested values of K ranging from 1 to 10. Because the right choice of K is a long-standing known issue with admixture modeling [41], we also performed a visual inspection of the results and presented more K values to describe population structure and substructures.

In addition, a network-based approach implemented in the program NetView v.1.1 [42, 43] using an Identity by State (IBS) distance matrix was performed to show relationships between genetic clusters inferred by Admixture. To visualize the population network connection for all the individuals, we used a k-NN value, a parameter determining the number of mutual nearest neighbors, selected by visual inspection of multiple k-NN values (k-NN = 10–60) plotted against the number of communities detected with a “Fast-greedy”, an “Infomap” and a “Walktrap” clustering algorithm (Supplementary Fig. S1).

Finally, relative migration between locations was estimated using the divMigrate function in the diveRsity package [44, 45]. Nei’s *G*_ST_ distances were calculated with 1,000 bootstrap replicates to detect significant asymmetrical gene flow between the sampled locations.

### Homozygosity and inbreeding

We assessed inbreeding using the estimate of the heterozygosity-fitness correlation (HFC). HFC relies on the general assumption that genome-wide heterozygosity is a good proxy for inbreeding because it reduces heterozygosity and generates identity disequilibrium (ID), i.e., correlation of heterozygosity across loci [46]. This is because inbred individuals are expected to be less heterozygous at all loci than outbred ones. We evaluated the ID by calculating the parameter g2 [47] using the inbreedR package [48]. The parameter g2 is an estimate of ID that measures the excess of standardised double heterozygosity at two loci relative to the expectation under random association (i.e., g2 = 0) [49]. Specifically, g2 estimates the variance in inbreeding and if it is significantly different from 0 it can be interpreted as a signature of HFC, thus inbreeding. The probability that g2 differs from zero (i.e., the null hypothesis of no variance in inbreeding) was assessed with 1,000 permutations and the standard error with 1,000 bootstraps.

Successively, to quantify autozygosity and inbreeding in the African buffalo in Mozambique, we estimated runs of homozygosity (ROH) using a sliding window method implemented in the R package DetectRuns [50]. For this analysis the following parameters were used: windowSize = 50, minSNP = 50, minDensity = 1/10,000, maxMissWindow = 1, maxGap = 1,000,000, minLengthBps = 1,000,000, maxOppWindow = 0. We explored the ROH pattern for each individual by plotting the number of ROH versus the total and mean ROH length (in Mbps) respectively. Successively we estimated the genome-wide inbreeding coefficient for each population based on the ROH (F_ROH_).

### Effective population size

We estimated historical and recent effective population size (N_e_) trajectories using two different approaches. First, we employed folded Site Frequency Spectrum (SFS) to infer the demographic history of African buffalo in Mozambique. We used Stairway Plot v2 [51], which employs a method that calculates the composite likelihood of the observed SFS under several possible size change scenarios across the history of a population. Stairway Plot estimates the most likely timing and magnitude of changes in *N*_*e*_ at various epochs, with the transition between epochs corresponding to coalescent events in the population. Stairway Plot calculates the population-scaled mutation rate θ, from which *N*_*e*_ can be derived. To do this, we use the µ (mutation rate) of ∼1.5 × 10^− 9^ estimated for ruminants [52] and a generation time of 7.5 years following previous studies of *S. c. caffer* historical demography [4, 5].

Subsequently, recent trends in N_e_ (below ∼1000 generations) were estimated using the program SNeP v1.1 [53], a multithreaded tool that assesses N_e_ based on linkage disequilibrium (LD). The method relies on the fact that LD between SNPs at different genetic distances provides information on N_e_ at different times in the recent past. We performed SNeP by using the standard PLINK file as input file format (.ped and.map files), considering all chromosomes in the analysis and by using Sved & Feldman [54] as recombination rate modifier. We also selected a MAF threshold of 0.05 because it has been demonstrated that accounting for MAF results in unbiased r^2^ estimates regardless of sample size [55].

## Results

### Genetic differentiation, population structure, admixture and gene flow

The average observed heterozygosity was lower than the expected heterozygosity in all the studied populations except for Gilè and Coutada 9 (Table 1), for which the estimate is based on only one individual each and thus cannot be considered sufficiently reliable. The remaining four populations show a comparable level of heterozygosity deficit and nucleotide diversity (Table 1). In contrast, the average inbreeding coefficient was higher in Marromeu and Gorongosa compared to the other populations. These two populations also exhibit the lowest values of genetic differentiation and absolute divergence when compared to Namaacha (Table 2).

**Table 1:** Number of individuals (N), Heterozygosity observed (H_o_) and expected (H_e_), nucleotide diversity (π), and inbreeding coefficient (F_is_). Buffalo populations except BC and BGI (both with *n* = 1) show a lower H_o_ than H_e_, a comparable level of nucleotide diversity. On the contrary, F_IS_ is particularly high in Gorongosa and Marromeu populations

Locality	N	H_o_	H_e_	π	F_is_
Coutada 9	1	0.00385 ± 0.0002	0.00192 ± 0.0001	0.0039 ± 0.0002	0
Catuane	3	0.00395 ± 0.0002	0.17297 ± 0.0007	0.2094 ± 0.00084	0.379 ± 0.0011
Gilè	1	0.00405 ± 0.0002	0.00203 ± 0.0001	0.0041 ± 0.0002	0
Gorongosa	26	0.00420 ± 0.0001	0.28581 ± 0.0005	0.2915 ± 0.0005	0.936 ± 0.0025
Marromeu	30	0.00407 ± 0.0001	0.28441 ± 0.0005	0.2895 ± 0.0005	0.926 ± 0.0045
Namaacha	9	0.00390 ± 0.0001	0.22539 ± 0.0006	0.2398 ± 0.0007	0.627 ± 0.0018

**Table 2:** Genomic differentiation (F_st_, above the diagonal) and absolute divergence (D_xy_, below the diagonal) are reported for the three populations with *n* > 9. Namaacha show the highest F_st_ respect to the other two populations. However, Dxy is only slightly higher

	Gorongosa	Marromeu	Namaacha
Gorongosa		0.022295	0.099829
Marromeu	0.002121		0.115915
Namaacha	0.002167	0.002211	

**Fig. 2:**
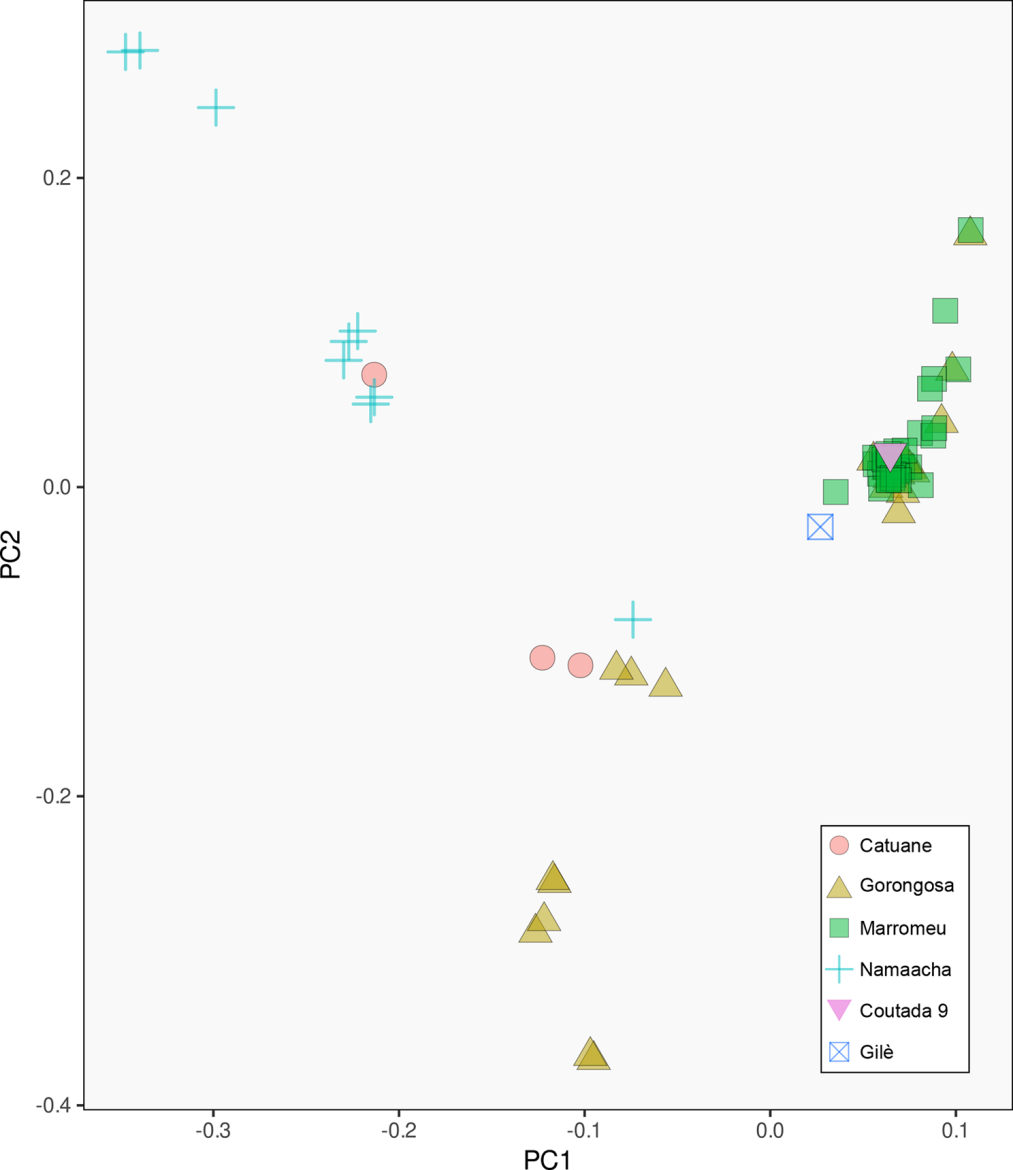
Principal Component Analysis (PCA) showing the presence of different genetic clusters within Mozambique. Marromeu is clearly distinct but largely overlaps with some individuals from Gorongosa, and it is also closely related to Gilè and Coutada 9. By contrast, individuals from Gorongosa form a second cluster that shows some relatedness with individuals from Namaacha and Catuane. Lastly, the remaining individuals from the southern locality form a third cluster

According to the PCA (Fig. 2), individuals from Marromeu cluster together showing low intrapopulation variability on the first two axes compared to other populations. In the same portion of the PCA space, we can find individuals from Gorongosa, Gilè, and Coutada 9. The remaining individuals from Gorongosa form a separate cluster. It is interesting to note that this second cluster includes two individuals from Catuane and one from Namaacha. Finally, the remaining individuals from Namaacha and Catuane form a distinguished group.

**Fig. 3:**
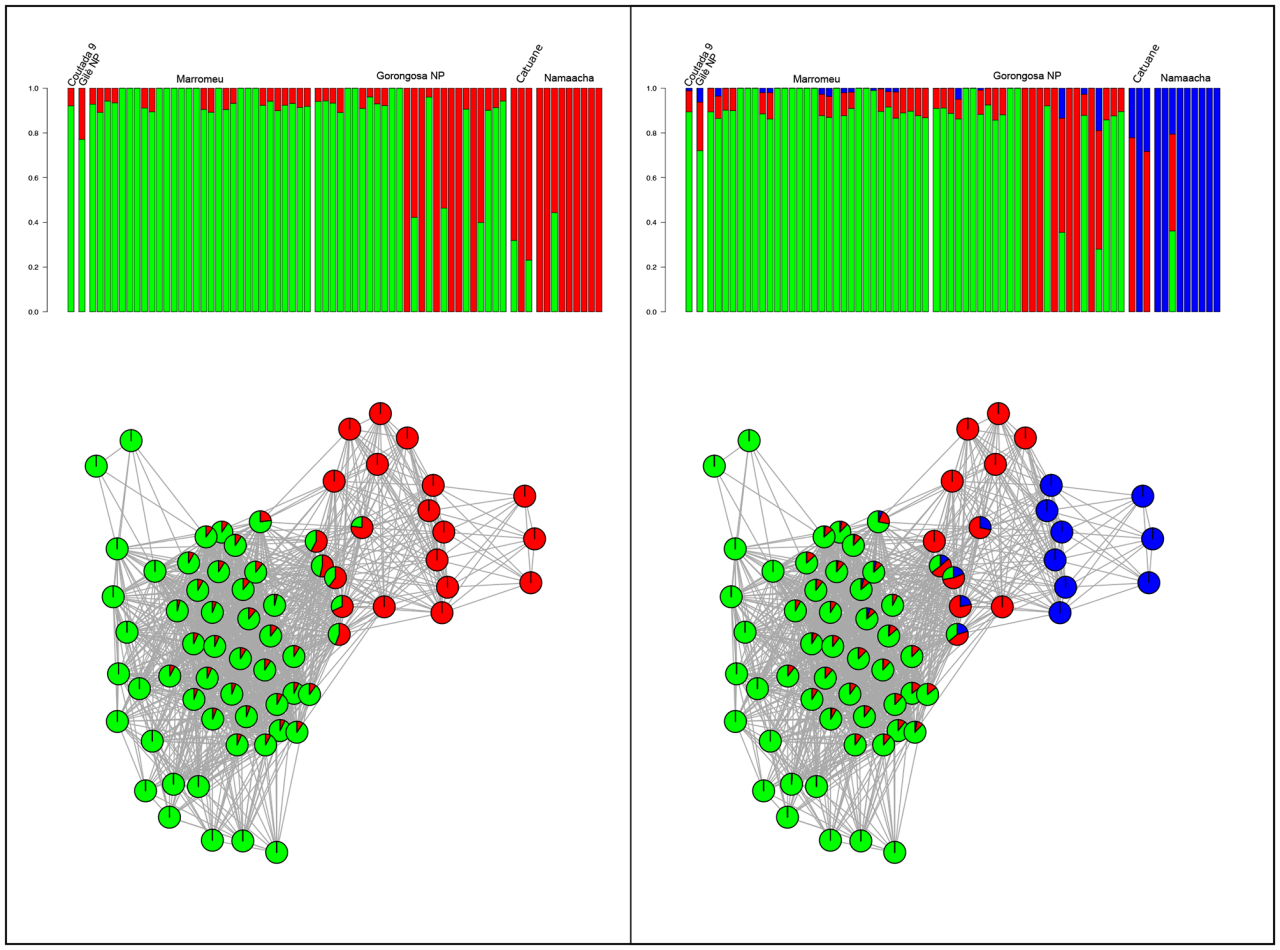
Admixture and network analysis. We reported the genetic clustering for K = 2 and K = 3, putative ancestral genotypes. For both K values, we can observe a clear structure of southern and central-northern individuals. Network analysis based on Identity by State distance matrix shows relationships between genetic clusters. Circles represent individuals whereas color represents cluster membership according to the Admixture analysis (K = 2 and K = 3 respectively)

In the Admixture analysis, the cross-validation (CV) shows an increasing error value from K = 1 to K = 10 (Supplementary Fig. S2). However, at K = 2 we observe just a very sharp increase of CV error compared to K = 1. By visually inspecting the admixture plots (Fig. 3) at K = 2, we observe a clear distinction between specimens from Namaacha and Catuane from specimens collected in Marromeu, Gilè, Coutada 9, and Gorongosa. However, nine individuals from Gorongosa are assigned to the other cluster or show a clear signature of admixture between the two clusters. At K = 3 (Fig. 3) individuals from Namaacha and one from Catuane form a new distinct genetic cluster. Three individuals from the southern clade still show admixture with the northern genetic clusters. This observation shows a congruence with the results depicted by the PCA (Fig. 2). Higher K values show a progressive increase of the cross-validation error (Supplementary Figure S2) and just a few differences can be observed within the Marromeu-Gorongosa cluster (data not shown).

The relationships among the clusters found by the Admixture analysis (K = 2 and K = 3) are summarised by the network shown in Fig. 3. The network shows that the admixed individuals found at K = 2 are effectively in an intermediate position. Interestingly, when cluster membership at K = 3 is plotted on the network, the third cluster identified by the Admixture analyses, mainly including individuals from Gorongosa and three other individuals from southern localities, occupies an intermediate position between the two main clusters.

**Fig. 4:**
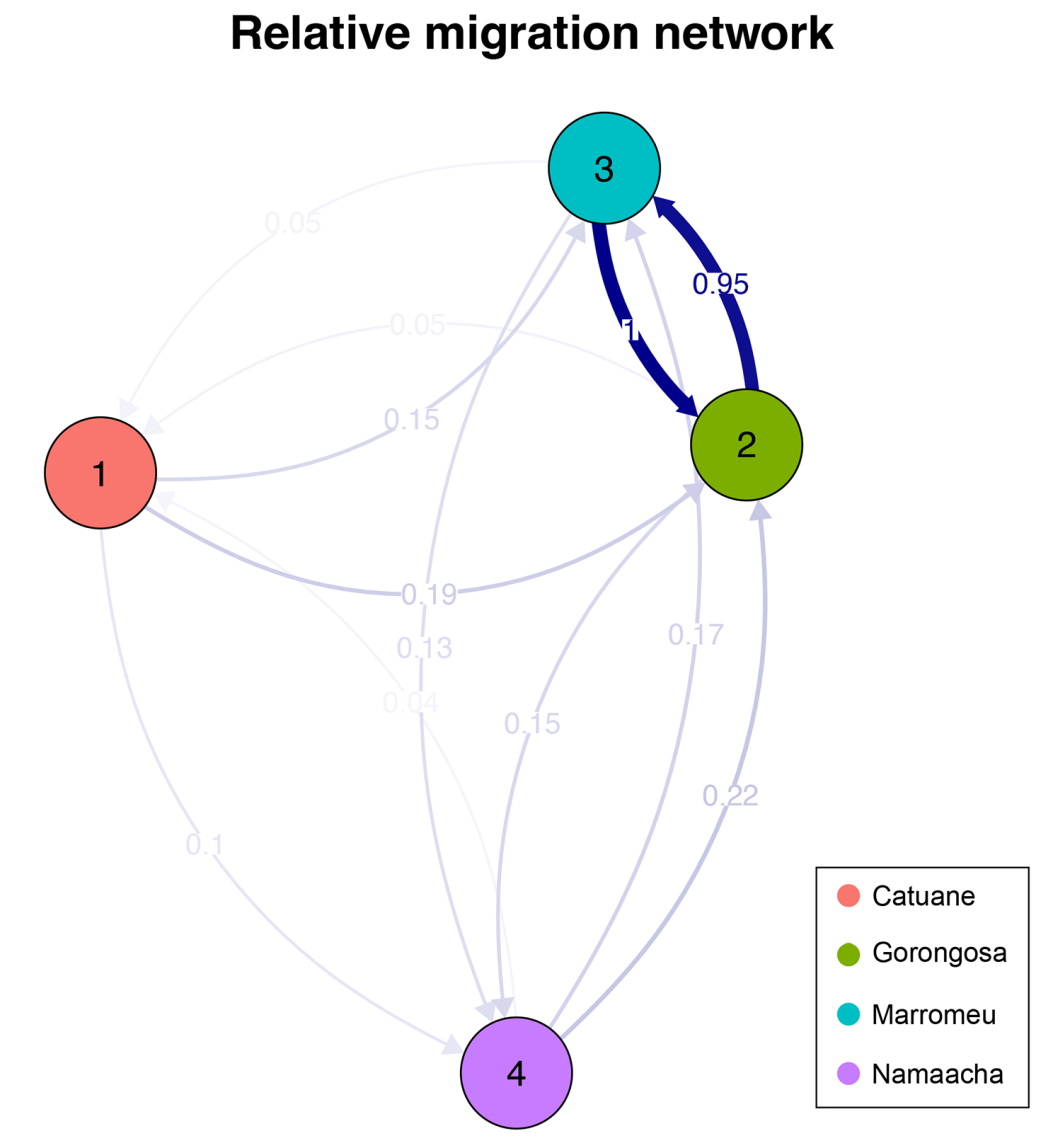
Among-population gene flow, estimated using Nei’s GST distances. The arrow’s color intensity is proportional to the level of gene flow. Only Gorongosa National Park and Marromeu Reserve show a high and symmetrical gene flow

As expected, the relative migration was found to be high and symmetrical between Gorongosa NP and Marromeu, whereas gene flow from south to north is very low in both directions (Fig. 4).

### Genome-wide homozygosity and inbreeding

Identity disequilibrium is low but positive and significantly different from 0 for all the analysed populations, giving a clear indication of HFC in all the populations. Catuane shows the lowest value (g2 = 4.89E-06; *p* = 0.001), followed by Gorongosa (g2 = 1.51E-04; *p* = 0.001) and Marromeu (g2 = 5.56E-04; *p* = 0.001), and by Namaacha (g2 = 3.70E-04; *p* = 0.001) with the highest values.

**Fig. 5:**
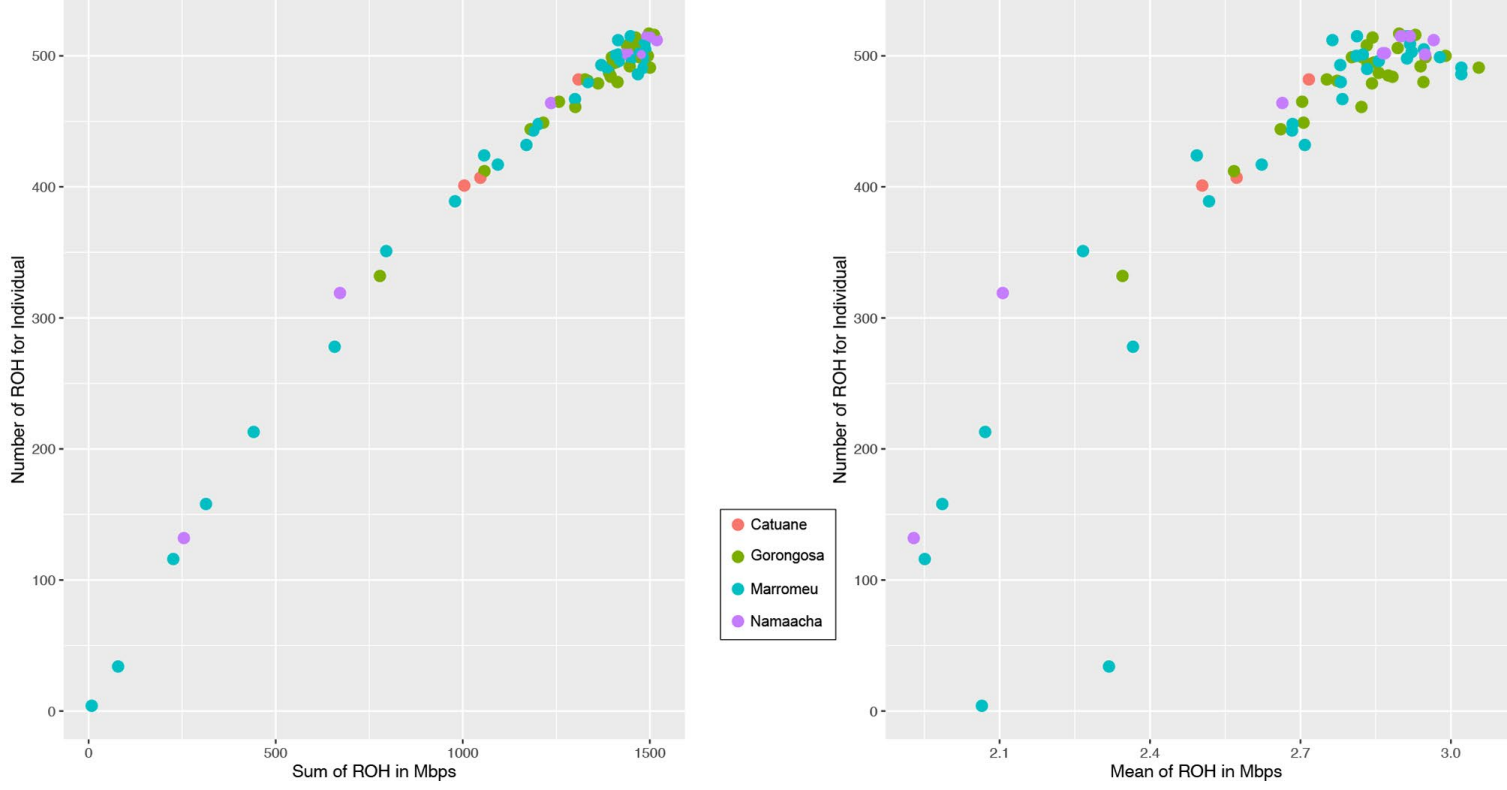
Distribution pattern of regions of homozygosity (ROH) length and number. Only a few individuals, mainly from Marromeu, show the presence of a ROH sum < 1000 Mb. The mean length of ROH ranges approximately from 2 to 3.5 Mb

The distribution patterns of regions of homozygosity (ROH) show that most of the analysed individuals show a high number (> 600) of long ROH regions (mean up to 3 Mbps, total length up to 1500 Mbps). Only a few individuals from Marromeu show a few short ROHs (Fig. 5).

**Fig. 6:**
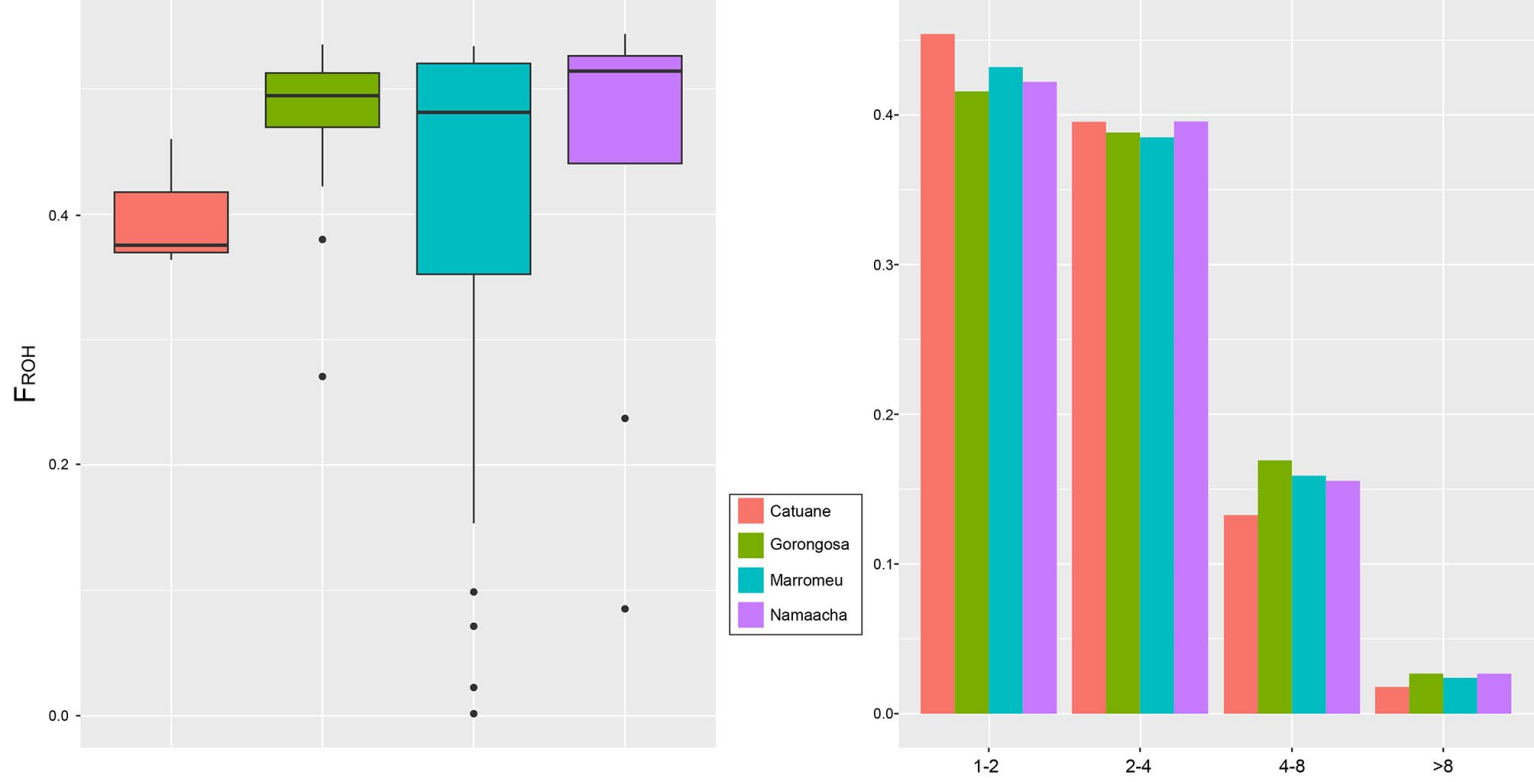
FROH coefficients (left) and distribution of ROH classes (right). All the populations analysed show a high FROH suggesting a large genome-wide homozygosity resulting from inbreeding. The lower and upper hinges correspond to the 25th and 75th percentiles. Lower and upper whiskers give a roughly 95% confidence interval around the median represented as a horizontal bold line. Pairwise t-test (with Bonferroni correction) reported no significant divergence between means (for summary statistics see supplementary Table S3). ROH graphs suggest the occurrence of a high percentage of short ROH (length < 2 Mb) in all populations, but also the presence of a significant fraction of ROHs > 4Mbp in length can be observed, indicating that most ROHs are the result of recent inbreeding events. Individual inbreeding coefficients are reported in Supplementary Table S2

These long stretches of homozygosity are reflected in a high level of inbreeding observed in all the populations (Fig. 6).

The frequency of ROH classes (Fig. 6) suggests the prevalence of large regions of long and very long ROH.

### Historical demography and effective population size estimation

**Fig. 7:**
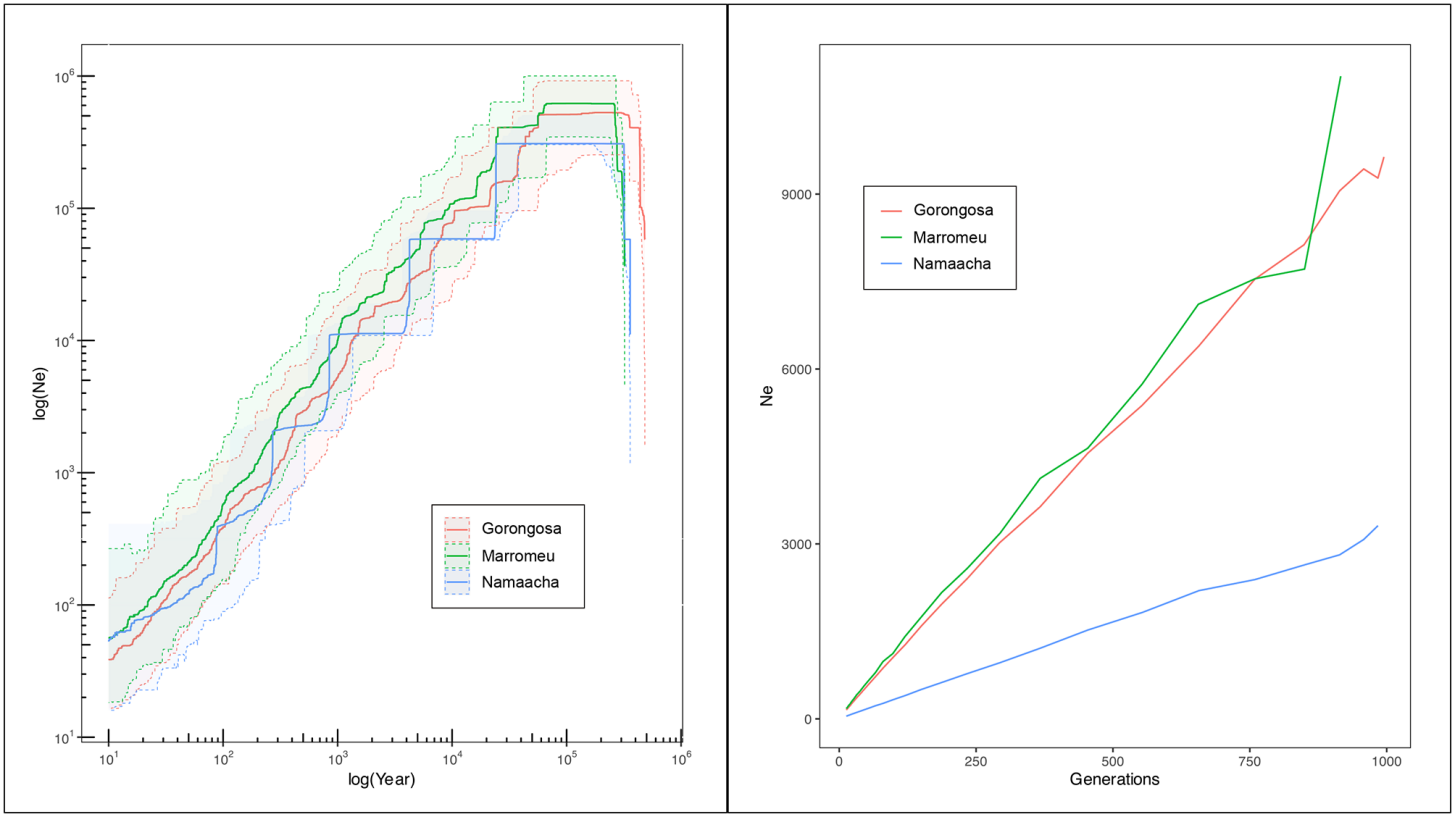
Effective population size estimates obtained for the Gorongosa, Marromeu, and Namaacha populations. Left panel: median N_e_ and 95% confidence intervals based on Site Frequency Spectrum (SFS). The three populations show a similar shape of N_e_ through time. Notably, all three populations show a N_e_ decrease starting at approximately around 50 ka. All three populations show a constant decrease of N_e_ until recent times without any signature of population expansion in recent times. Right panel: the recent N_e_ trend obtained using linkage disequilibrium in SNeP software is coherent with what we observed using the SFS method. All the populations show a N_e_ decrease also in recent times reaching a very low N_e_ (see Table 3)

Finally, the plot of N_e_ over time (Fig. 7) shows a clear decline for all the populations analysed (Catuane, Gilè, and Coutada 9 were excluded due to the very low sample size). Following a stationary phase that started approximately 300–350 thousand years ago (ka), all three populations show a N_e_ decrease starting approximately between 30 and 50 ka (Fig. 7). For all three populations the N_e_ decrease was constant until recent time, without any signature of population expansion in recent times. According to SNeP estimates, the last 1000 generations were characterised by a steep decrease (Fig. 7). Both methods provide coherent final estimates with a N_e_ a few decades of individuals (Table 3).

**Table 3:** Current effective population size. The Table shows the final estimates of N_e_ obtained using two different methods: a median estimate of N_e_ with a 95% confidence interval was obtained using SFS in the Stairway plot 2. A recent N_e_ estimate obtained using linkage disequilibrium in SNeP

Population	N_e_ Stairway Plot 2	N_e_ SNeP
Gorongosa	11.31 (1.13–87.15)	153
Marromeu	34.11 (3.13-243.84)	176
Namaacha	38.98 (5.00-410.13)	46

## Discussion

Our findings provide new data on the conservation status and genetic diversity of some African buffalo populations from Mozambique. As expected, we observed evidence of genetic variability loss, reduced gene flow, and excess of homozygosity in a large part of the genome. Additionally, we found evidence of past connections between southern and northern populations. This information may aid in the planning of further studies and assist in the management of surviving buffalo herds.

### Population structure and admixture in Mozambique African buffaloes

Historical data indicates that buffalo populations in Mozambique, as well as other large herbivores, were abundant at the onset of the colonial period [2]. However, since the end of the 19th century the increase of human settlement, the hunting pressure, and the expansion of cattle have resulted in the demographic decline of many large herbivores including *S. caffer* [2]. More recently, buffalo populations in Mozambique have undergone a dramatic numerical reduction due to persecution during the civil war that ended in 1992. Currently, the African buffalo primarily survives in protected areas with herds ranging from a few dozen to a few thousand individuals. Our study found these areas are weakly connected by gene flow, with the only exception of Marromeu and Gorongosa. Consequently, *S. c. caffer* in Mozambique exhibits a significant population structure, which is consistent with previous studies based on microsatellite data [7]. An interesting finding in our study is the unexpected population substructure observed in Gorongosa. While most individuals from Gorongosa showed genetic similarity to nearby Marromeu, a few individuals exhibited clear attribution to the southern cluster or evidence of admixture between southern and northern clusters. At K = 3 (three genetic clusters), these individuals are clearly distinct from both southern and northern clades, and according to the network analysis, they represent an intermediate genotype that connects southern and northern Mozambique buffaloes. Therefore, the Gorongosa population displays an unexpected population substructure with one of the two genotypes closely related to Marromeu, due to their geographical proximity, while the second cluster is apparently more related to southern individuals (as evidenced by the presence of two individuals from Catuane). Differently from previous studies conducted on buffaloes from the same area [7], we analysed autochthonous individuals from Gorongosa that survived human persecution during the civil war and were not recently introduced from South Africa. Based on this, one possible explanation of the presence of an unexpected genetic cluster in Gorongosa is that in the past the genetic connectivity between southern and northern herds was higher than it is now, and Gorongosa served as a biogeographical hinge between southern and northern Mozambique. In contrast, all the other populations analysed appear to be genetically homogeneous, likely due to the range restriction of all herds in Mozambique to a few protected and managed areas, resulting in minimal gene flow, especially in the last century.

### Historical and contemporary demographic trends

In our analysis, the three distinct populations exhibit remarkably similar trends in effective population size (N_e_), implying the occurrence of comparable drivers of demographic decline across Mozambique. This decline started roughly between 40 and 50 ka and persisted until the present era determining a very low N_e_. These findings display partial alignment with recent literature. Indeed, De Jager et al. [4], employing a Sequentially Markovian Coalescent (PMSC) method, identified a signature of N_e_ decline within the South African *S. c. caffer* population around 50 ka, a pattern corroborated by our results from Stairway Plot 2. Notably, De Jager et al. [4] documented an expansion event around 30 ka, which contrasts with our observations. Subsequently, Quinn et al. [5], adopting an Approximate Bayesian Computation (ABC) simulation, identified a continuous N_e_ decline in South African population since roughly 10 ka, mirroring our recent demographic trajectory estimate obtained through SNeP and Stairway Plot 2. Presuming that both the Mozambique and South African buffalo populations were subject to analogous or at least similar drivers of demographic changes, the absence of a detected expansion around 30 ka could be partly attributed to methodological disparities. It is worth noting that the methodologies employed in our study, along with the ABC simulation method used by Quinn et al. [5], exhibit better performance in capturing recent historical demography when compared to the PMSC method [51, 56, 57]. Consequently, we are inclined to assert that our estimation of a recent and sustained decline is a reliable representation.

Our estimation of the demographic decline finds parallelism with a significant reduction in population and concurrent species extinctions documented in Africa since the late Pleistocene, where approximately 24 large mammal species are known to have disappeared from the continent during the last ∼100,000 years [58]. The precise causal factors behind these extinctions remain a subject of ongoing debate [58, 59]. However, a mounting body of evidence suggests that the disappearance of numerous large herbivores, likely preceded by declines in population density, can be explained by an interplay of natural and anthropogenic variables. These include shifts in climate, alterations in the environment, species interactions, and the expansion of human populations [60–62]. Hence, we argue that the observed decline in the population of *S. c. caffer* started from the latter stages of the Pleistocene could similarly be attributed to these phenomena. Certainly, for more recent population declines, human impact emerges as the principal cause. In the case of buffalo populations within the southern African region, historical events such as European colonization increased hunting pressure but also facilitated the transmission of diseases from domesticated cattle to wild herbivores [62]. In the specific context of Mozambique, the buffalo populations were dramatically affected by the civil war. Before this conflict, the Marromeu and Gorongosa area harbored some of the largest buffalo herds in southern Africa, with an estimated population of around 50,000 and 14,000 individuals. However, in the aftermath of the civil war, this population experienced an impressive contraction, leaving an estimated 2,500 buffaloes remaining in the Marromeu region and approximately less than 100 individuals in Gorongosa [11, 63]. Although the demographic crisis in Marromeu was not as severe as in Gorongosa, it still resulted in a significant erosion of genetic diversity. This is evident from low heterozygosity and PCA and Admixture analysis, which show that Marromeu individuals are genetically homogeneous. Thus, all the past and recent perturbations discussed above may have had a profound influence on the survival of buffalo populations in Mozambique and collectively, the convergence of these factors could explain our estimation of a continuous N_e_ decline since 50 ka and the remarkably low effective population size estimate observed in this study.

### Genome-wide homozygosity and inbreeding

As expected, the observed N_e_ trends were accompanied by a heterozygosity deficit and a pervasive presence of ROH stretches in all the studied populations. Our sample revealed that only a few individuals had a low number of short ROHs, while the majority had many long ROHs. It is well established that the occurrence of long and large ROHs is related to the demographic history of populations, and the ROH pattern observed in our sample is expected for populations that have experienced recent inbreeding and bottleneck [64, 65]. Elevated levels of inbreeding have been previously documented within *S. c. caffer* populations in South Africa [4, 5]. Specifically, Quinn et al. [5] identified pronounced inbreeding within the southernmost populations, namely Hluhluwe-iMfolozi Park and Addo Elephant National Park, characterised by a notable abundance of long ROH. These extended ROH segments were attributed to recent inbreeding events, particularly impacting these two populations. In contrast, within Mozambique, all buffalo populations exhibit approximately 30% of ROH segments exceeding 4 megabases (Mb) in length, with the cumulative length of individual ROHs exceeding that observed in many South African populations but more closely aligned with the high levels of inbreeding noted in the inbred populations of Hluhluwe-Imfolozi Park and Addo Elephant National Park [5]. Instances of high levels of the fraction of the genome in *runs of homozygosity* (measured as F_ROH_) have been recently reported for the Indian tiger [66] and in African for black rhinos from South Africa [67], as well as the plain zebra populations in East Africa [68]. In these cases, the shared characteristics are small population sizes, geographic isolation, and the relatively recent occurrence of inbreeding, culminating in an excessive frequency of extended ROH segments. Therefore, the pronounced prevalence of ROHs observed within Mozambique’s buffalo population, which exceeds the levels observed in South African counterparts and mirrors those seen in other populations of critically endangered species, indicates recent and pervasive inbreeding in Mozambique. Both the utilised metrics, g2, and F_ROH_, consistently indicate a comparable degree of inbreeding across all populations. The co-occurrence of such elevated F_ROH_ values and statistically significant g2 signals points towards a scenario wherein an excess burden of recessive deleterious alleles is likely present. This, in turn, could trigger a fitness depression within the population.

### Implication for management and conservation

From a conservation genetic perspective, the widespread presence of large and numerous ROHs is a cause for concern as it can have a dramatic impact on the survival of *S. c. caffer* in Mozambique. Studies on the wild Soay sheep have shown that individuals with long ROHs are more likely to succumb during their first winter, owing to the high frequency of deleterious mutations in these regions [69]. In the African buffalo, van Hooft et al. [31] highlighted the occurrence of a latitudinal cline of recessive alleles, which might negatively affect male body condition and bovine tuberculosis resistance. This suggests that a massive loss of animals in Mozambique cannot be ruled out, especially in the face of new diseases or environmental changes. In addition, the possibility of pathogens being introduced along with translocated individuals is a real risk [70], and the effect on a genetically depauperate population is hard to predict. Furthermore, due to the lack of corridors for gene flow, the progeny of introduced individuals could be susceptible to genetic diversity loss due to founder effects and small population size [71]. All these factors could lead to inbreeding depression and, ultimately, jeopardize the success of any translocation effort in the long run. Finally, it is interesting to note that van Hooft et al. [31, 72] also found that male-deleterious alleles could be epigenetically suppressed in a large fraction of animals. Therefore, future work focused on the management and conservation of buffalo populations should also explore epigenetic mutations as potential markers for past and present environmental stress events, as well as indicators of individual physiological conditions [73]. This information has the potential to enhance conservation plans by considering the capacity of organisms to rapidly adapt to environmental changes, thus improving the conservation of wild populations [73].

## Conclusions

We found evidence of *S. c. caffer* decline in Mozambique dating back 100,000 years. This decline is congruent with previous studies focusing on the African buffalo in the South African region. According to our data, this decline persisted until recent times, culminating in a contemporary N_e_ estimate that is very low, as expected for populations that have experienced conflict with human activities and habitat loss. The observed loss of genetic diversity could expose the species to vulnerability to pathogens and possibly local extinction. The African buffalo plays a crucial role as an ecosystem engineer by preparing habitat for other herbivores. Therefore, preserving robust and resilient buffalo populations is vital to maintain a functional ecosystem. One potential solution to reduce long genomic regions in homozygosity is to introduce animals from compatible populations to increase genetic diversity. Despite successful reintroduction in Mozambique, such as in Gorongosa National Park, our findings suggest that buffaloes from Mozambique, including those from southern localities that likely originated from South Africa [74], have suffered from genetic erosion and possess long and abundant ROHs. The widespread inbreeding within *S. c. caffer*, also outside Mozambique [5], with potential source populations characterised by a high level of homozygosity, hence possibly a carrier of genetic load of recessive alleles, represents a significant issue that can determine the success or failure of any translocation program and should be taken seriously into consideration. We recommend that future translocation efforts should consider this aspect and conduct a survey of genome-wide homozygosity in all potential donor populations before initiating a restocking intervention. This will increase the likelihood of success in preserving genetic diversity and maintaining a functional ecosystem.

### Electronic supplementary material

Below is the link to the electronic supplementary material.

Supplementary Table S1: Specimen ID, sampling locality and sampling date of all the individuals used.

## Data Availability

All sequence data presented here (*fastq* format) have been deposited into the NCBI SRA database (accession number: PRJNA971211).
