# Supplementary material for: Genome-wide diversity, population structure and signatures of inbreeding in the African buffalo in Mozambique
Source: BMC Ecol Evol. 2024 Mar 4;24:29. doi: 10.1186/s12862-024-02209-2 (PMC10910738; doi:10.1186/s12862-024-02209-2)
Supplement: Supplementary file 1 — Supplementary Table S1: Specimen ID, sampling locality and sampling date of all the individuals used. [file 12862_2024_2209_MOESM1_ESM.pdf]

Supplementary files

Genome-wide diversity, population structure and signatures of inbreeding in the African buffalo in Mozambique

Paolo Colangelo[1], Marika Di Civita[2,3], Carlos M. Bento[4], Paolo Franchini[5,6], Axel Meyer[5], Nadiya Orel[5], Luis C. B. G. das Neves[7,8], Fernando C. Mulandane[7], Joao S. Almeida[9], Gabriele Senczuk[2], Fabio Pilla[2], Simone Sabatelli[3]

[1] National Research Council, Research Institute on Terrestrial Ecosystems, Via Salaria km 29.300, 00015 Montelibretti (Roma), Italy

[2] University of Molise, Department of Agricultural, Environmental and Food Sciences, 86100 Campobasso, Italy

[3] Sapienza University, Department of Biology and Biotechnologies “Charles Darwin”, Via A. Borelli 50, 00161, Roma, Italy

[4] Natural History Museum, Eduardo Mondlane University, Travessia do Zambeze 104, Maputo 1100, Mozambique

[5] Department of Biology, University of Konstanz, Konstanz, Germany

[6] Department of Ecological and Biological Sciences, University of Tuscia. Viale dell’Università s.n.c, 01100 Viterbo, Italy

[7] Biotechnology Centre of Eduardo Mondlane University, Maputo, Mozambique

[8] Department of Veterinary Tropical Diseases, Faculty of Veterinary Sciences, University of Pretoria, Pretoria, South Africa

[9] Mozambique wildlife alliance, Maputo, Mozambique.

Supplementary Table S1: Specimen ID, sampling locality and sampling date of all the individuals used.

N.	Specimen ID	Locality	Coordinates	Sampling Date
1	BGP1	Gorongosa National Park	18°54'54" S 034°29'01" E	2007
2	BGP2	Gorongosa National Park	18°54'54" S 034°29'01" E	2007
3	BGP3	Gorongosa National Park	18°54'54" S 034°29'01" E	2007
4	BGP5	Gorongosa National Park	18°54'54" S 034°29'01" E	2007
5	BGP7	Gorongosa National Park	18°54'54" S 034°29'01" E	2007
6	BGP8	Gorongosa National Park	18°54'54" S 034°29'01" E	2007
7	BGP12	Gorongosa National Park	18°54'54" S 034°29'01" E	2007
8	BGP13	Gorongosa National Park	18°54'54" S 034°29'01" E	2007
9	BGP14	Gorongosa National Park	18°54'54" S 034°29'01" E	2007
10	BGP16	Gorongosa National Park	18°54'54" S 034°29'01" E	2007
11	BGP17	Gorongosa National Park	18°54'54" S 034°29'01" E	2007
12	BGP18	Gorongosa National Park	18°54'54" S 034°29'01" E	2007
13	BGP19	Gorongosa National Park	18°54'54" S 034°29'01" E	2007
14	BGP20	Gorongosa National Park	18°54'54" S 034°29'01" E	2007
15	BGP21	Gorongosa National Park	18°54'54" S 034°29'01" E	2007
16	BGP22	Gorongosa National Park	18°54'54" S 034°29'01" E	2007
17	BGP23	Gorongosa National Park	18°54'54" S 034°29'01" E	2007
18	BGP25	Gorongosa National Park	18°54'54" S 034°29'01" E	2007
19	BGP27	Gorongosa National Park	18°54'54" S 034°29'01" E	2007
20	BGP28	Gorongosa National Park	18°54'54" S 034°29'01" E	2007

21	BGP30	Gorongosa National Park	18°54'54" S 034°29'01" E	2007
22	BGP31	Gorongosa National Park	18°54'54" S 034°29'01" E	2007
23	BGP32	Gorongosa National Park	18°54'54" S 034°29'01" E	2007
24	BGP33	Gorongosa National Park	18°54'54" S 034°29'01" E	2007
25	BGP40	Gorongosa National Park	18°54'54" S 034°29'01" E	2007
26	BGP47	Gorongosa National Park	18°54'54" S 034°29'01" E	2007
27	BMR3	Marromeu National Reserve	18°50'16" S 035°51'48" E	2017
28	BMR4	Marromeu National Reserve	18°50'16" S 035°51'48" E	2017
29	BMR5	Marromeu National Reserve	18°50'16" S 035°51'48" E	2017
30	BMR6	Marromeu National Reserve	18°50'16" S 035°51'48" E	2017
31	BMR7	Marromeu National Reserve	18°50'16" S 035°51'48" E	2017
32	BMR8	Marromeu National Reserve	18°50'16" S 035°51'48" E	2017
33	BMR9	Marromeu National Reserve	18°50'16" S 035°51'48" E	2017
34	BMR10	Marromeu National Reserve	18°50'16" S 035°51'48" E	2017
35	BMR11	Marromeu National Reserve	18°50'16" S 035°51'48" E	2017
36	BMR12	Marromeu National Reserve	18°50'16" S 035°51'48" E	2017
37	BMR13	Marromeu National Reserve	18°50'16" S 035°51'48" E	2017
38	BMR14	Marromeu National Reserve	18°42'16" S 035°51'48" E	2017
39	BMR15	Marromeu National Reserve	18°42'16" S 035°51'48" E	2017
40	BMR16	Marromeu National Reserve	18°42'16" S 035°51'48" E	2017
41	BMR17	Marromeu National Reserve	18°42'16" S 035°51'48" E	2017
42	BMR18	Marromeu National Reserve	18°42'16" S 035°51'48" E	2017
43	BMR19	Marromeu National Reserve	18°42'16" S 035°51'48" E	2017

44	BMR20	Marromeu National Reserve	18°42'16" S 035°51'48" E	2017
45	BMR21	Marromeu National Reserve	18°42'16" S 035°51'48" E	2017
46	BMR23	Marromeu National Reserve	18°42'16" S 035°51'48" E	2017
47	BMR24	Marromeu National Reserve	18°42'16" S 035°51'48" E	2017
48	BMR25	Marromeu National Reserve	18°42'16" S 035°51'48" E	2017
49	BMR26	Marromeu National Reserve	18°42'16" S 035°51'48" E	2017
50	BMR27	Marromeu National Reserve	18°42'16" S 035°51'48" E	2017
51	BMR28	Marromeu National Reserve	18°42'16" S 035°51'48" E	2017
52	BMR30	Marromeu National Reserve	18°42'16" S 035°51'48" E	2017
53	BMR34	Marromeu National Reserve	18°42'16" S 035°51'48" E	2017
54	BMR35	Marromeu National Reserve	18°42'16" S 035°51'48" E	2017
55	BMR36	Marromeu National Reserve	18°50'16" S 035°51'48" E	2017
56	BMR37	Marromeu National Reserve	18°50'16" S 035°51'48" E	2017
57	BNA3	Namaacha	25°57'48" S 032°29'01" E	2019
58	BNA4	Namaacha	25°57'48" S 032°29'01" E	2019
59	BNA5	Namaacha	25°57'48" S 032°29'01" E	2019
60	BNA7	Namaacha	25°57'48" S 032°29'01" E	2019
61	BNA8	Namaacha	25°57'48" S 032°29'01" E	2019
62	BNA9	Namaacha	25°57'48" S 032°29'01" E	2019
63	BNA12	Namaacha	25°57'48" S 032°29'01" E	2019
64	BNA14	Namaacha	25°57'48" S 032°29'01" E	2019
65	BNA16	Namaacha	25°57'48" S 032°29'01" E	2019
66	BC9.1	Coutada 9 -Manica	17°34'51" S 033°37'30" E	2019

67	BGI1	Gilé Reserve	16°34'06" S 038°24'31" E	2019
68	BCAT1	Catuane	26°51'02" S 032°17'07" E	2019
69	BCAT2	Catuane	26°51'02" S 032°17'07" E	2019
70	BCAT3	Catuane	26°51'02" S 032°17'07" E	2019

Supplementary Table S2. Individual F_{ROH} inbreeding coefficient for all the specimens. We also reported the locality and the sum of ROH.

id	Locality	ROH [sum]	F_{ROH}
BC9	Coutada 9	1290533371	0.498
BCAT1	Catuane	1192150238	0.460
BCAT2	Catuane	942358354	0.364
BCAT3	Catuane	972587073	0.375
BGI1	Gilé Reserve	1257483280	0.485
BGP1	Gorongosa National Park	1283233148	0.495
BGP12	Gorongosa National Park	1356187794	0.523
BGP13	Gorongosa National Park	1266284134	0.489
BGP14	Gorongosa National Park	1215292154	0.469
BGP16	Gorongosa National Park	1127490248	0.435
BGP17	Gorongosa National Park	1297578918	0.501
BGP18	Gorongosa National Park	1189556314	0.459
BGP19	Gorongosa National Park	1262174755	0.487
BGP2	Gorongosa National Park	1311608327	0.506
BGP20	Gorongosa National Park	1322761109	0.510
BGP21	Gorongosa National Park	1330369890	0.513
BGP22	Gorongosa National Park	1349708446	0.521
BGP23	Gorongosa National Park	701240721	0.271
BGP25	Gorongosa National Park	1387413055	0.535
BGP27	Gorongosa National Park	1273980482	0.492
BGP28	Gorongosa National Park	1151950175	0.445
BGP3	Gorongosa National Park	1295208329	0.500
BGP30	Gorongosa National Park	1094217189	0.422
BGP31	Gorongosa National Park	984413243	0.380
BGP32	Gorongosa National Park	1278552678	0.493
BGP33	Gorongosa National Park	1382447360	0.533
BGP40	Gorongosa National Park	1319944852	0.509
BGP47	Gorongosa National Park	1220408944	0.471

BGP5	Gorongosa National Park	1366883670	0.527
BGP7	Gorongosa National Park	1280096683	0.494
BGP8	Gorongosa National Park	1370432232	0.529
BMR10	Marromeu National Reserve	1350904843	0.521
BMR11	Marromeu National Reserve	1340541365	0.517
BMR12	Marromeu National Reserve	556516574	0.215
BMR13	Marromeu National Reserve	1273606307	0.491
BMR14	Marromeu National Reserve	1353618207	0.522
BMR15	Marromeu National Reserve	1353086854	0.522
BMR16	Marromeu National Reserve	1383864407	0.534
BMR17	Marromeu National Reserve	1070397708	0.413
BMR18	Marromeu National Reserve	1366255302	0.527
BMR19	Marromeu National Reserve	397936361	0.154
BMR20	Marromeu National Reserve	255911785	0.099
BMR21	Marromeu National Reserve	1281351595	0.494
BMR23	Marromeu National Reserve	1310634720	0.506
BMR24	Marromeu National Reserve	1371087891	0.529
BMR25	Marromeu National Reserve	185016437	0.071
BMR26	Marromeu National Reserve	1319170670	0.509
BMR27	Marromeu National Reserve	1010529744	0.390
BMR28	Marromeu National Reserve	1083675449	0.418
BMR3	Marromeu National Reserve	967044332	0.373
BMR30	Marromeu National Reserve	1199420527	0.463
BMR34	Marromeu National Reserve	1380648132	0.533
BMR35	Marromeu National Reserve	1260546931	0.486
BMR36	Marromeu National Reserve	58437595	0.023
BMR37	Marromeu National Reserve	894367747	0.345
BMR4	Marromeu National Reserve	690840994	0.267
BMR5	Marromeu National Reserve	1357400502	0.524
BMR6	Marromeu National Reserve	1112341291	0.429
BMR7	Marromeu National Reserve	1279290454	0.494
BMR8	Marromeu National Reserve	1233887471	0.476

BMR9	Marromeu National Reserve	4341660	0.002
BNA12	Namaacha	1363996127	0.526
BNA14	Namaacha	1409198387	0.544
BNA16	Namaacha	1362992906	0.526
BNA3	Namaacha	614641297	0.237
BNA4	Namaacha	221018073	0.085
BNA5	Namaacha	1141589005	0.441
BNA7	Namaacha	1385936235	0.535
BNA8	Namaacha	1325254736	0.511
BNA9	Namaacha	1332541321	0.514

Supplementary Table S3: Mean and median F_{ROH}, with variance and standard deviation for each Locality/Population (see also Figure 6 in the main text)

Population	Mean F_{ROH}	Median F_{ROH}	Variance of F_{ROH}	Standard Deviation of F_{ROH}
Countada 9	0.4980	0.4980		
Catuane	0.3997	0.3753	0.0028	0.0526
Gilé Reserve	0.4852	0.4852		
Gorongosa National Park	0.4811	0.4946	0.0032	0.0567
Marromeu National Reserve	0.3949	0.4813	0.0285	0.1687
Namaacha	0.4355	0.5142	0.0265	0.1628

Supplementary Figure S1

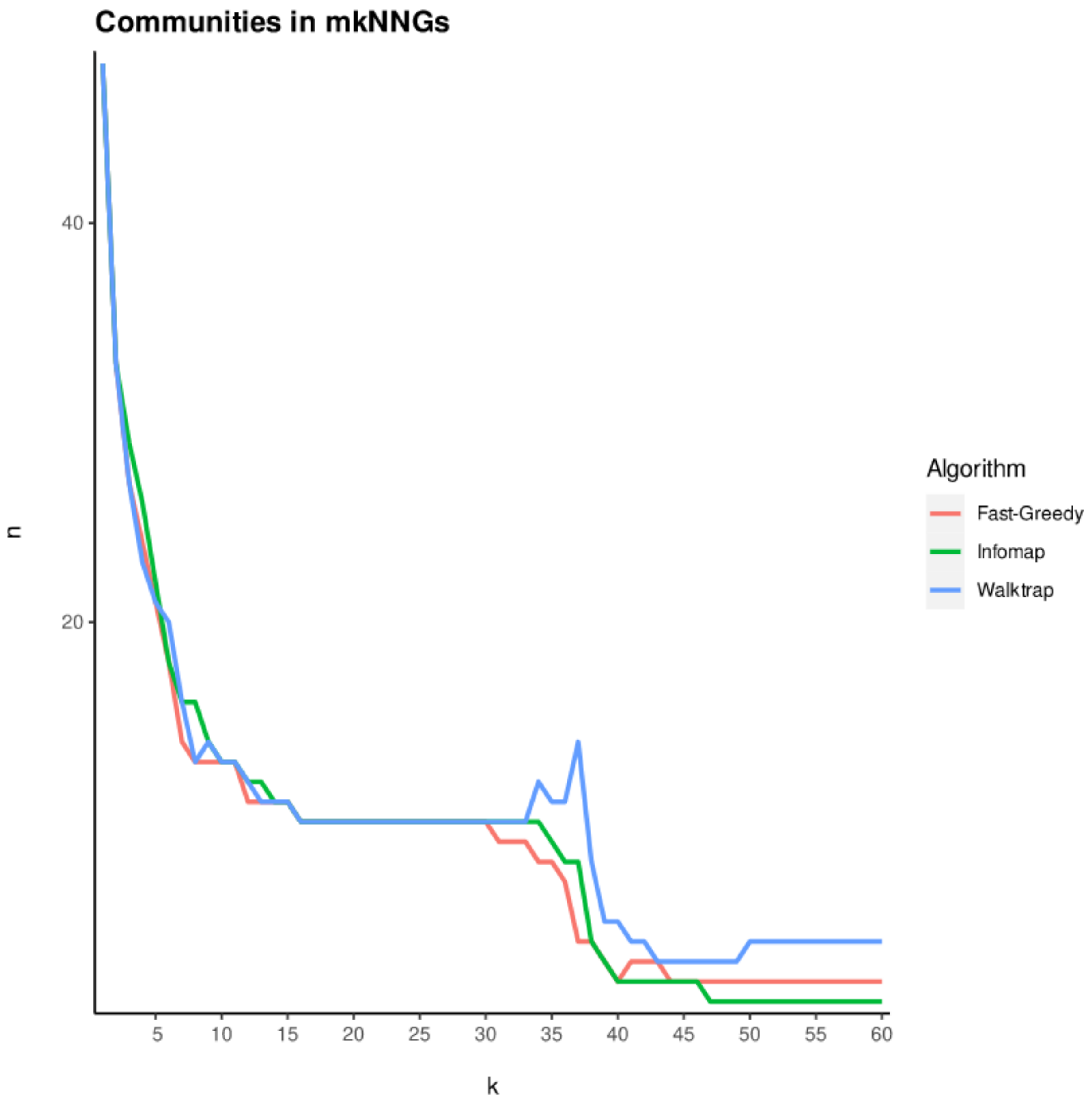

Supplementary Figure S1. k-NN values plotted against the number of communities detected using a “Fast-greedy”, an “Infomap” and a “Walktrap” clustering algorithm.

Supplementary Figure S2

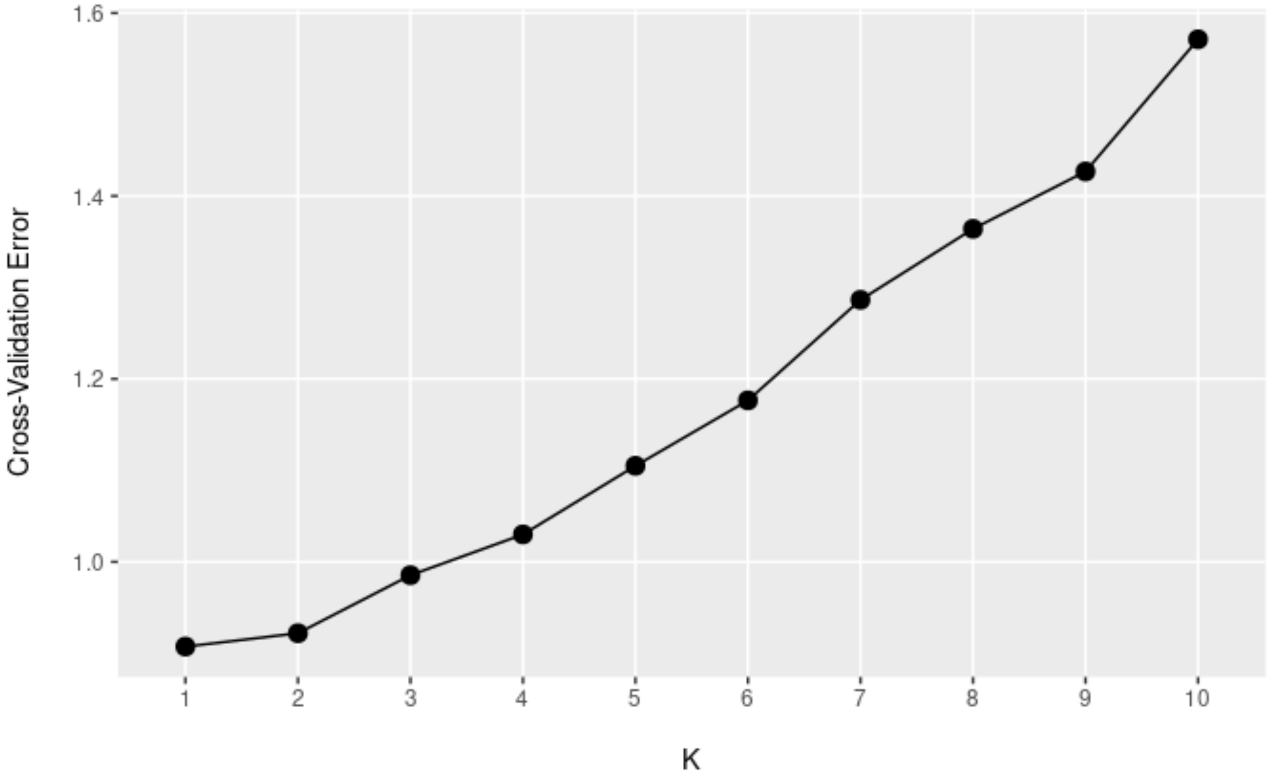

Supplementary Figure S2. Cross Validation error (CVE) for the Admixture analysis (5-fold CV). We explored K from 1 to 10. K=1 and K=2 show almost identical values (CVE=0.91 and 0.94 respectively).
